# Ferroptosis programmed cell death in AD‐derived neurons

**DOI:** 10.1002/alz.089791

**Published:** 2025-01-03

**Authors:** German Plascencia‐Villa, George Perry

**Affiliations:** ^1^ The University of Texas at San Antonio (UTSA), San Antonio, TX USA; ^2^ The University of Texas at San Antonio, San Antonio, TX USA

## Abstract

**Background:**

Neurodegeneration is characterized by the progressive loss of neurons. However, the mechanisms by which neurons die in Alzheimer’s disease (AD) remain elusive. Disrupted iron homeostasis is associated with accelerated cognitive decline, amyloid beta deposition, and AD progression, but its pathogenic relevance is poorly understood. The recent discovery of ferroptosis (an iron‐dependent regulated cell death mechanism) provides an alternative potential explanation for the relevance or iron dysregulation in AD.

**Method:**

AD‐derived neurons are used to determine neurotoxicity, morphological alterations and understanding of ferroptotic responses and progression. Neurons were dosed with ferroptosis‐inducing agents to determine susceptibility to oxidative stress and cellular responses.

**Result:**

We performed oxidative stress assays to quantify glutathione (GSH/GSSG), reactive oxygen species (ROS), NAD/NADPH, lipid peroxidation, mitochondrial dysfunction and intracellular iron deposition in AD‐derived neurons. Furthermore, the neuronal responses to oxidative stress‐ferroptosis is visualized with live‐cell holotomography (quantitative phase microscopy) to determine the neuronal ferroptotic phenotype.

**Conclusion:**

Understand the role of iron accumulation, oxidative stress, and cell‐specific susceptibility to ferroptosis programmed cell death in human brain cells and model the complex molecular responses related to neuronal loss in AD. These experiments provide the kinetics of biochemical, metabolic and morphological features of AD neurons under ferroptotic oxidative stress.

